# ISOBAR Implementation for Patient Handover Between Rescue Services and Pediatric Emergency Department Staff: The COPTER-PED Trial

**DOI:** 10.1016/j.acepjo.2025.100300

**Published:** 2025-12-06

**Authors:** Vincent Bietke, Sebastian Lang, Tabea Maass, Thomas Lehmann, Birgitta Hucke, Melanie Rohmann, Hans Proquitté, Jan-Christoph Lewejohann, Matthias Nuernberger

**Affiliations:** 1 Department of Emergency Medicine, Jena University Hospital, Friedrich Schiller University, Jena, Germany; 2 Department for Anesthesiology and Intensive Care Medicine, Jena University Hospital, Friedrich Schiller University, Jena, Germany; 3 Centre for Clinical Studies (ZKS), Jena University Hospital, Friedrich Schiller University, Jena, Germany; 4 Department of Pediatrics, Jena University Hospital, Friedrich Schiller University, Jena, Germany

**Keywords:** pediatric emergency medicine, pediatric emergency department, interdisciplinary communication, patient safety, ISOBAR, patient handoff, patient handover

## Abstract

**Objectives:**

Miscommunication is a major contributing factor to adverse events in medical care, highlighting the importance of thorough patient handover—especially in complex settings like pediatric emergency medicine. This study evaluates the effects of adopting the ISOBAR handover protocol for transfers from emergency medical services to pediatric emergency department (PED) staff.

**Methods:**

We conducted a single-center implementation trial to evaluate the ISOBAR handover protocol’s efficacy in a German university hospital’s PED. The procedure was based on the previous COPTER study. We observed 107 handovers, comparing those conducted using the ISOBAR protocol with those following standard procedure without a protocol. The trial was organized into 4 consecutive phases, alternating between interventional periods. The primary outcome measure was the “Key Information Transfer Efficiency” (KITE) score. The secondary outcome measure was how PED personnel retained key information after the handover.

**Results:**

By the end of the study, the amount of key information transferred during the handover had increased by 22%. PED physicians remembered 16% and PED nurses 10% more key information. KITE reflected the course of the implementation. However, full adherence to the ISOBAR protocol did not have a significant effect on the outcome measures.

**Conclusion:**

Although the use of ISOBAR can improve the exchange of information during handover in the PED, strict adherence to the protocol is not essential. What really matters is high-quality communication throughout the entire patient handover process.

**The Bottom Line**

When emergency medical services bring children to the hospital, important details can easily be lost during the handover. We assessed the simple communication tool ISOBAR to structure the information exchanged between emergency medical services and pediatric emergency department personnel. Overseeing 107 handovers with intervention periods alternating between ISOBAR-guided and nonguided, the use of ISOBAR improved the efficiency of information exchange and the information retention of pediatric emergency department staff. Physicians remembered 16% and nurses 10% more relevant facts. Strict adherence to the ISOBAR framework however was less important than maintaining concise, focused communication.

## 1 Introduction

### 1.1 Background

Miscommunication is a leading cause of adverse events in medical care,^1^, ^2^, ^3^ often linked to inadequate quality of information exchanged between individuals.^4^ Improving handover quality, therefore, is relevant for patient safety.^5^, ^6^, ^7^ In a previous study in our adult emergency department (ED)^8^, the implementation of the ISOBAR protocol improved communication: staff recalled 20% more relevant information after the handover. In pediatric emergency medicine, handover quality is equally important, especially due to the high variability of cases and the limited routine among emergency medical services (EMS). Most children are brought to the pediatric emergency department (PED) by their parents^9^, ^10^, ^11^; whereas, EMS is called mainly for rare but severe emergencies, ranging from neonatal seizures to adolescent intoxications. Only 1 or 2 EMS-to-PED handovers occur daily in our hospital, limiting opportunities for routine. Children’s restricted ability to describe symptoms, reliance on nonverbal cues, and the emotional stress experienced by patients, families, and staff make communication especially challenging and increase the risk of information loss during transfer.^12^^,^^13^

### 1.2 Importance

Structured handover protocols, such as ISOBAR reduce information loss in principle, especially in complex cases or unfamiliar situations.^14^^,^^15^ ISOBAR can close information gaps and reduce adverse events.^2^^,^^16^, ^17^, ^18^, ^19^ Its concise structure suits emergency medicine, where handovers must be swift and focused. Although studies on specific protocols for the PED (eg, ED I-PASS^20^) have shown benefits, the effect of the popular and universal tool ISOBAR has not yet been thoroughly investigated.

### 1.3 Goals of This Investigation

This study evaluated the impact of the ISOBAR framework in a German PED, adapted from the previous adult COPTER trial.^8^ We hypothesized that ISOBAR would make handovers briefer and more focused, improving information retention among PED staff.

## 2 Methods

### 2.1 Design and Setting

**Figure 1:**
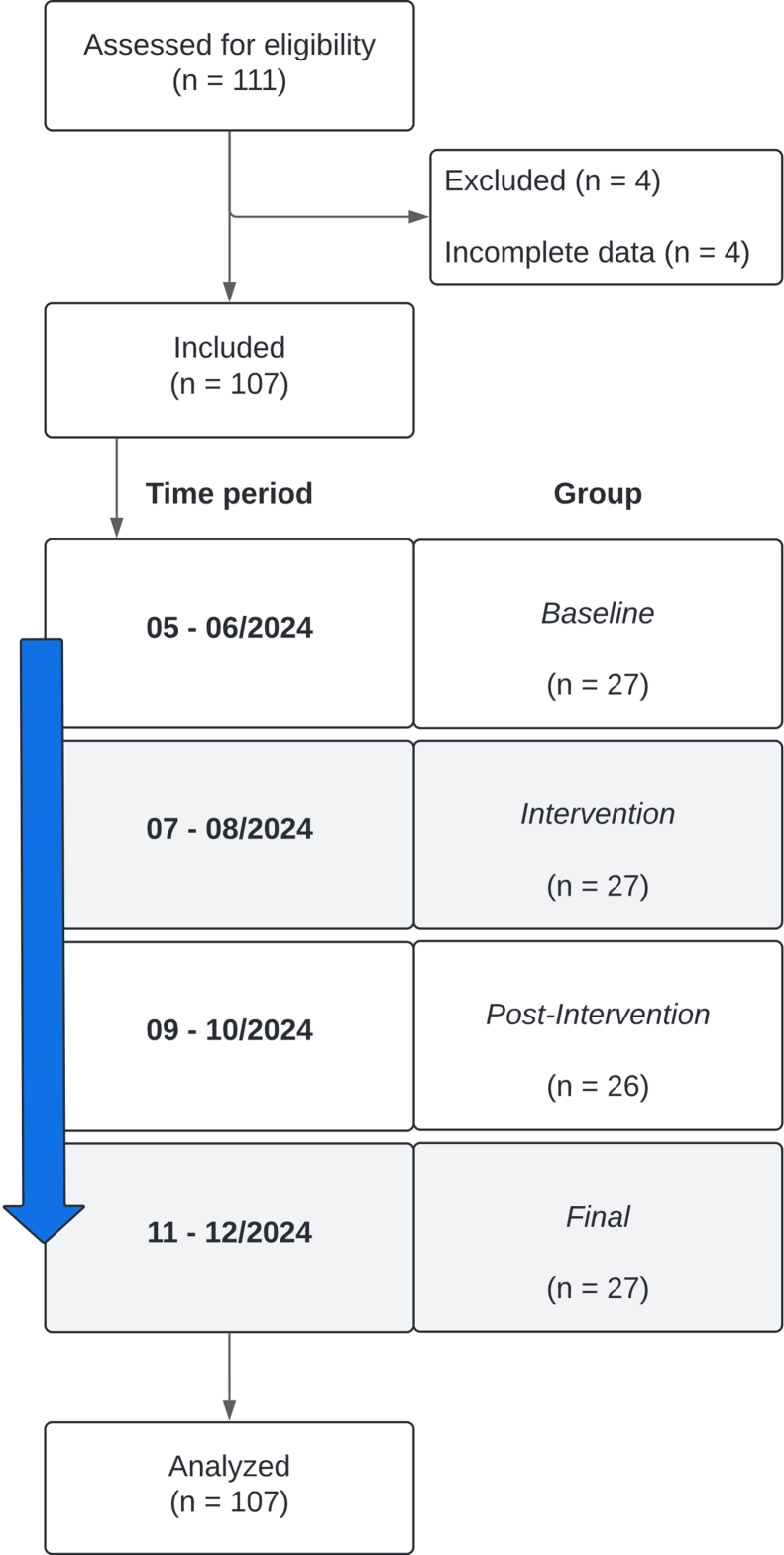
Flowchart of study procedure: time interval on the left and study group (including number of handovers) on the right, including the CONSORT flowchart.

The present study was designed as a single-center implementation trial and carried out in the PED of a German university hospital, treating about 9000 pediatric patients annually. The methodology mirrored the previous COPTER trial in our adult ED.^8^ Our PED and ED operate independently with separate staff and locations. Both are served by the same EMS teams. We observed 111 EMS-to-PED handovers between May and December 2024 (Fig 1). The study followed CONSORT^21^ and StaRI^22^ guidelines, was approved by the local ethics committee (No. 2024-3274-BO), registered in the German Clinical Trials Registry (ID: DRKS00031223), and included a pilot trial (*n* = 8).

### 2.2 Selection of Subjects

All EMS-to-PED handovers during the study period were eligible. Our PED receives 1 or 2 EMS presentations per day. Inclusion occurred daily between 9 AM and 9 PM during 2-hour observation blocks. In total, 111 handovers were observed, and 4 were excluded due to incomplete data. Allocation followed a time-sequenced design across 4 consecutive phases (Fig 1), resulting in 4 trial groups: *baseline*, *intervention*, *postintervention*, and *final*. See for more information in Supplementary Appendix 1.1.

### 2.3 Intervention

The intervention consisted of the stepwise implementation of ISOBAR across 4 consecutive phases (Fig 1). No formal training was provided; changes in handover practice were induced by visual aids and verbal prompts. During *baseline*, handovers were nonguided. During *intervention*, ISOBAR was introduced through departmental policy, supported by a schematic poster and onsite reminders. In *postintervention*, both posters and prompts were removed. *Final* reinstated the poster, added larger EMS-area visual aids, and resumed reminders.

### 2.4 Measures

**Figure 2:**
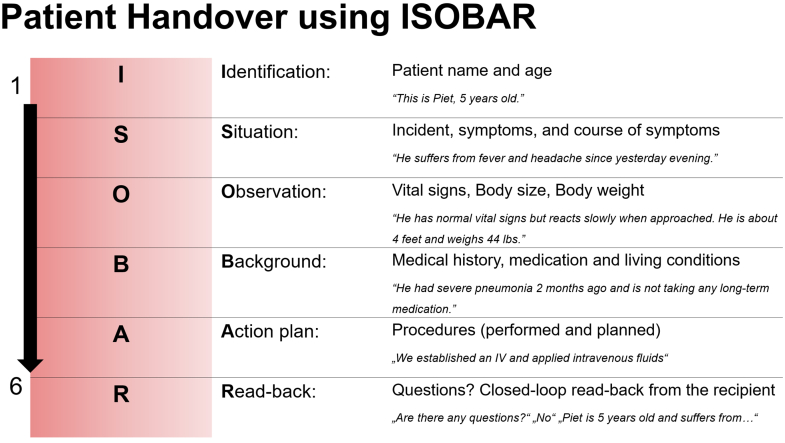
ISOBAR protocol for patient transfer communication. Each letter of the acronym refers to key information. Example in italics illustrates intended content.

All measures mirrored those in the previous COPTER trial.^8^ ISOBAR was adapted for pediatric needs by including height, weight, and relatives (Fig 2). Forms were adapted for the PED as well (Figs S1, S2, and S3). All data were verified by a research coordinator and stored in a secure REDCap database.^23^ See for more information in Supplementary Appendix 1.2.

### 2.5 Outcomes

All outcomes mirrored those in the previous COPTER trial.^8^ The primary outcome was the Key Information Transfer Efficiency (KITE) score. The secondary outcome was the amount of information retained by PED staff in a standardized interview 15 minutes after the handover. See for more information in Supplementary Appendix 1.3.

### 2.6 Data Analysis

Sample size was determined a priori based on the pilot trial. Data analysis was performed in the same way as in the previous trial. We used an alpha level of 0.05 to determine significance See for more information in Supplementary Appendix 1.4.

## 3 Results

### 3.1 Group Characteristics and Comparisons

No significant differences were found between the 4 groups regarding workload, nurses’ experience, or case complexity (*P* > .05). Physicians’ experience changed from a mean of 5.25 years (95% CI, 3.55-6.96) in *baseline* to 3.33 (95% CI, 2.00-4.67) in *final* (*P* = .015). ISOBAR adherence rates were 48% in *intervention*, 58% in *postintervention*, and 52% in *final*.

### 3.2 Primary Outcome Measure: KITE

**Figure 3:**
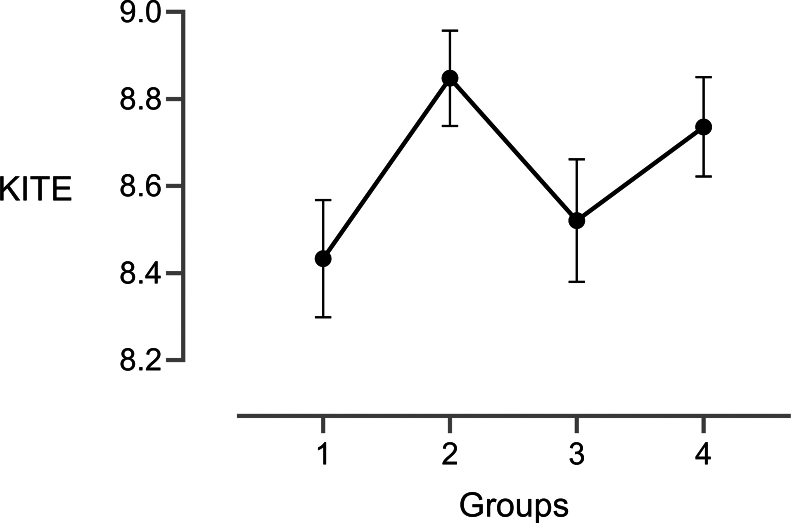
Key Information Transfer Efficiency (KITE) (mean ± 95% CI) for all 4 groups across the whole trial duration. 1, baseline; 2, intervention; 3, postintervention; and 4, final.

**Figure 4:**
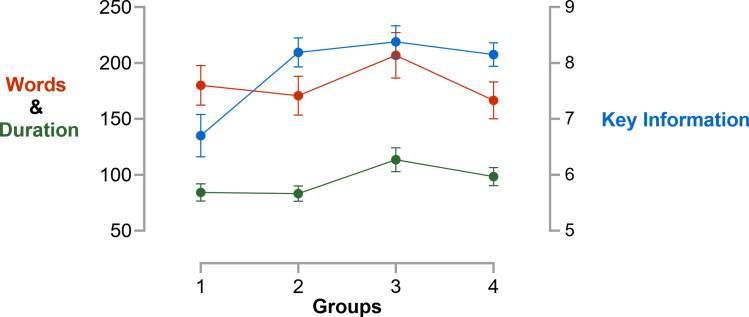
Words (orange), duration (green, in seconds), and conveyed key information (blue, *Y*-axis on the right) in patient handover conversations across the course of the study (mean ± SEM). *X*-axis shows the 4 groups (ie, time clusters) of the study. SEM of duration was small and is not shown. 1, baseline; 2, intervention; 3, postintervention; and 4, final.

KITE scores increased significantly from *baseline* (mean 8.43; 95% CI, 8.16-8.71) to *intervention* (mean 8.84; 95% CI, 8.62-9.07; *P* = .032), declined slightly in *postintervention* (mean 8.52; 95% CI, 8.23-8.81; *P* = .41), and rose again in *final* (mean 8.74; 95% CI, 8.50-8.97; *P* = .113), see Figure 3. Between *baseline* and *final*, word count (167 vs 180, *P* = .775) and duration (98 vs 84 seconds, *P* = .18) did not differ, but key information improved markedly (6.70 vs 8.15, *P* = .004, +21.64%), see Figure 4 and Table S1.

### 3.3 Secondary Outcome Measure: Key Information Retention

**Figure 5:**
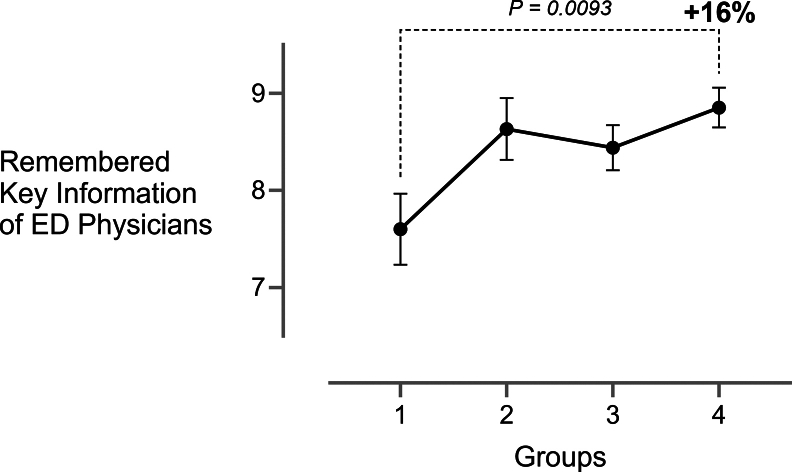
Remembered key information of the pediatric emergency department physician 15 minutes after patient handover (mean ± 95% CI) for all 4 groups across the whole trial duration. For clarity, only the comparison between baseline and final is shown. 1, baseline; 2, intervention; 3, postintervention; and 4, final. ED, emergency department.

**Figure 6:**
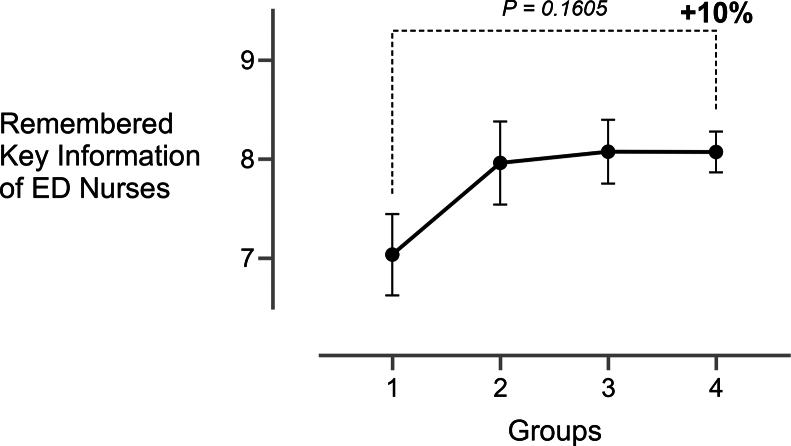
Remembered key information of the pediatric emergency department nurse between 5 and 25 minutes after patient handover (mean ± 95% CI) for all 4 groups across the whole trial duration. For clarity, only the comparison between baseline and final is shown. 1, baseline; 2, intervention; 3, postintervention; and 4, final. ED, emergency department.

Physicians’ recall improved significantly from *baseline* (mean 7.60; 95% CI, 6.83-8.37) to *intervention* (mean 8.63; 95% CI, 7.97-9.30; *P* = .029) and *final* (mean 8.85; 95% CI, 8.43-9.27; *P* = .005), representing a 16 % increase in remembered key information (Fig 5). Nurses’ recall rose by about 10 % from *baseline* (mean 7.31; 95% CI, 6.64-7.97) to *intervention* (mean 8.27; 95% CI, 7.66-8.88; *P* = .02) but remained the same afterward (Fig 6). The number of key information transmitted correlated positively with information retention for nurses (*ρ* = .42, *P* < .001) and physicians (*ρ* = .39, *P* < .001).

### 3.4 Intervention Impact: Subjective Rating

*Final* handover ratings were better than *baseline*, indicating a trend toward improved perceived handover quality (physicians, 1.80 vs 1.67; *P* = .681; nurses, 2.00 vs 1.89; *P* = .645).

### 3.5 ISOBAR-Adherence

ISOBAR adherence did not significantly affect KITE scores (*P* = .642) or information recall (*P* = .668 for nurses; *P* = .419 for physicians). The interaction effect between group and ISOBAR adherence did not reach statistical significance, *P* = .805.

### 3.6 Inquiries

**Figure 7:**
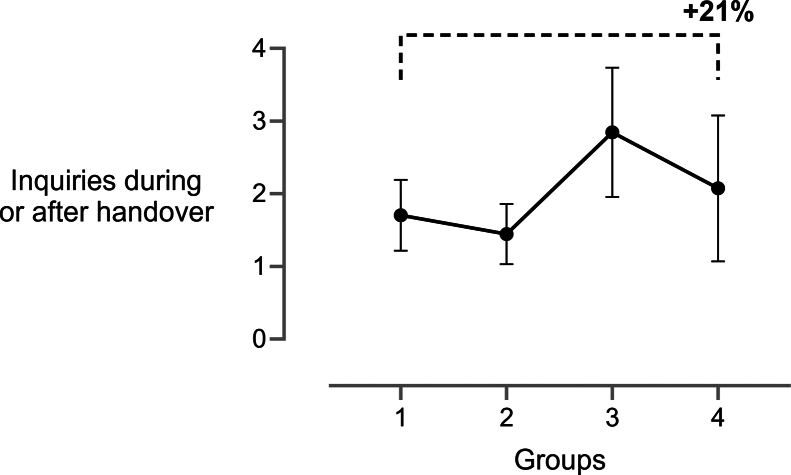
Inquiries (questions) during or after patient handover (mean ± 95% CI) for all 4 groups across the whole trial duration. For clarity, only the comparison between baseline and final is shown. 1, baseline; 2, intervention; 3, postintervention; and 4, final.

An increase in posthandover questions was observed, peaking in *postintervention* (mean 2.85; 95% CI, 1.95-3.74) compared with *baseline* (mean 1.70; 95% CI, 1.22-2.91; *P* = .048; mean change, 1.15) and *intervention* (mean 1.44; 95% CI, 1.03-1.86; *P* = .011; mean change, 1.41) (Fig 7).

## 4 Limitations

This study shares limitations with the previous COPTER trial,^8^ especially possible yet unavoidable performance and participant bias. We implemented the same controlling techniques as before but cannot rule out residual effects. The staggered design approach is common for implementation trials but may have added to participant bias. The design was selected for procedural reasons, as it offers the best balance between implementation and daily routine work.

Our local EMS teams serve both ED and PED. This might have introduced familiarization with ISOBAR during the preceding trial. After the previous study, no visual indicators remained in place in our adult ED. Yet, the prior exposure may have resulted in a more structured handover style at baseline in the present study, reducing the visible contrast between the intervention phases. Because the same EMS teams were active in all study phases, such an influence would have been consistent and would not likely have caused systematic bias. Inclusion was limited to about 14 handovers per month due to the low number of EMS-to-PED transfers (1-2 per day), restricting the sample but reflecting reality.

## 5 Discussion

Our findings confirm that implementing the ISOBAR framework in the PED significantly improved information transfer, reflected especially in greater recall among staff. These results parallel those of our earlier COPTER trial.^8^ Differences between settings, however, are notable. During this study, physicians’ average experience decreased between phases while nurses’ experience remained stable. Information transfer improved despite this decline, suggesting that structured handovers particularly benefit less experienced physicians. Nurses also showed marked gains, likely due to their central role in pediatric care. Pediatric-specific elements such as weight and height were increasingly included, underscoring both the adaptability and the relevance of tailoring ISOBAR.

This study provides important insights into structured handovers: although information transfer improved significantly, strict adherence to the newly introduced protocol was not the only decisive factor. Not only do these findings repeat those of the previous study, but they also confirm their transferability: the effects occur in the complex field of pediatric emergency medicine as well. The previously prevailing assumption that maximum implementation fidelity leads to optimal results is being called into question. Instead, it can be considered whether communication quality is more important than rigid compliance. Handover tools can be reinterpreted, moving away from stiff checklists toward flexible cognitive frameworks.

The results help to resolve the adherence paradox.^24^ This refers to a critical implementation problem: high protocol adherence and substantial improvements in communication clarity occur predominantly in the classroom, whereas only small to moderate effects were observed in clinical settings. Lo et al.^24^ recommended that future implementation efforts should first confirm high adherence rather than assuming it. However, the present study shows that protocol implementation without strict adherence can achieve significant improvements when the focus is on communication during handover.

## Author Contributions

Study concept and design: VB, SL, TM, TL, BH, MR, HP, J-CL, MN.

Acquisition of data: VB, MN.

Analysis and interpretation of the data: VB, TM, BH, MR, HP, MN.

Drafting: VB, MN.

Revision: TL, SL, BH, MR, HP, J-CL, MN.

Statistical expertise: VB, TL, MN.

Acquisition of funding: MN.

## Funding and Support

Advanced Clinician Scientist Program, Interdisciplinary Center of Clinical Research of the Medical Faculty Jena, ACSP10. Open Access funding is enabled and organized by Project DEAL.

## Conflict of Interest

All authors have affirmed they have no conflicts of interest to declare.
