# Supplementary material for: ISOBAR Implementation for Patient Handover Between Rescue Services and Pediatric Emergency Department Staff: The COPTER-PED Trial
Source: J Am Coll Emerg Physicians Open. 2025 Dec 6;7(1):100300. doi: 10.1016/j.acepjo.2025.100300 (PMC12723129; doi:10.1016/j.acepjo.2025.100300)
Supplement: Supplementary Material [file mmc1.docx]

# Supplementary Material

## Time-Sequenced Design

This trial’s design included two time-sequence implementation research methods. First, the procedure of the study was divided into four time periods, which defined four study groups. The length of these periods was chosen based on seasons and the expected number of PED patients compared to previous years, hence matching the study groups. The allocation of a patient to a group therefore depended on the date of presentation. This design combines the consideration of temporal trends with a pragmatic approach, whereby the representativeness of the points in time is maintained. We chose this study design to support the external validity of our outcome measures. Second, the inclusion occurred daily between 9 am and 9 pm for two hours. The exact time for these two hours was determined at the beginning of the study: a two-hour block was randomly assigned to each day of the month. This allocation was the same for every day and every month for the entire duration of the study. The two-hours blocks were: 9-11 am, 11 am-1 pm, 1-3 pm, 3-5 pm, 5-7 pm, and 7-9 pm. For example: On every fourth day of each month, admission took place between 3 and 5 pm. Blocks were allocated to no more than 6 days a month.

EMS included emergency physicians, paramedics, and technicians; PED included physicians and nurses involved in handover as well as follow-up interviews. Exclusion criteria were Emergency Severity Index (ESI) category 1 or severe pain (Numerical Rating Scale ≥ 8). ESI 1 is extremely rare in our PED (2-3 per year), and these patients are treated in our adult ED due to space requirements. No ESI 1 occurred during the study period.

## Measures

Each patient handover was observed by two trained research assistants and the principal investigator. Due to privacy protection regulations recordings were not permitted. The research assistants used standardized collection forms (Supplementary Figures S1 & S2) to collect data: key information provided, the sequence of that information, total word count, handover duration, questions raised during handover, and adherence to the ISOBAR framework meaning the correct sequence. Key information is relevant information for a patient’s treatment. Word counts were recorded manually using calibrated mechanical hand counters. The assistants underwent prior training to ensure consistent counting accuracy and performed periodic cross-checks to maintain inter-observer agreement above 95%. Handover duration was measured with digital stopwatches, starting when the EMS began the report and ending when the PED team confirmed completion. Fifteen minutes after each handover, PED staff were interviewed using a standardized form to assess retention of conveyed information (Supplementary Figure S3).

## Outcomes

The KITE score integrates the number of words, the duration in seconds, and the number of key information items transmitted. A high KITE score indicates efficient information transfer during handover. It is inversely related to the number of words and duration: a higher word count and a longer conversation duration led to a lower score, while a larger number of key information items increase the score. The KITE score has been used in the previous COPTER study,^1^ which demonstrated its applicability. Further methodological details of KITE development are available in the Supporting Information of the cited publication.

Fifteen minutes after each handover, research assistants conducted standardized interviews with PED staff to assess which key information was still recalled. The 15-minute interval reflects the optimal span for assessing short-term memory. Staff also rated handover quality (1 = very good to 6 = insufficient), case complexity (low, medium, high), and their own professional experience in years.

Research assistants documented potential disruptive factors, including staff shortages, handovers outside designated areas, background noise, time pressure, and interruptions (each coded yes/no). These variables were combined into a composite measure representing the current workload.

| **KITE Calculation Formula:** |
| --- |
| KITE (Z) = *key information (Z, log10)* $\times$ $\frac{1}{duration (Z, log10)}$ $\times$ $\frac{1}{word count (Z, log10)}$  KITE (Z10) = *KITE* $\times$ $\frac{10}{KITE Z max}$ |

## Sample Size and Statistical Analysis

Data was analyzed in SPSS Statistics 29.0.0 (IBM). Figures were generated with Prism 10.2.1 (GraphPad Software, LLC.).

Based on data from the pilot trial, we expected a mean KITE of 8.39 (SD 0.15). A t-test for log-normal distributed data with 26 patients per group was projected to provide more than 80% power to detect an effect at a 5% significance level, assuming the intervention group’s results are at least 1.2 times greater than the control group. Total sample size was therefore determined to be 26 per group, amounting to 104 in total.

Normality was assessed using the Shapiro-Wilk test. We employed the Mann-Whitney U test for comparing two independent groups. The effect sizes was calculated using eta squared (η2), which represents the proportion of variance explained by the factor. Eta squared was derived from the H statistic with its respective degrees of freedom (df). We used the Kruskal-Wallis test for group differences among all four groups. Effect sizes are given as Eta-squared (η^2^). Homogeneity of variances was assessed using Levene’s test without correction.

Multiple linear regression models during KITE development were fitted for continuous outcomes with different independent variables and standardized regression coefficients are reported with 95% confidence intervals.

# Supplementary Figures

## Standardized collection form – Patient Handover (Form 1):


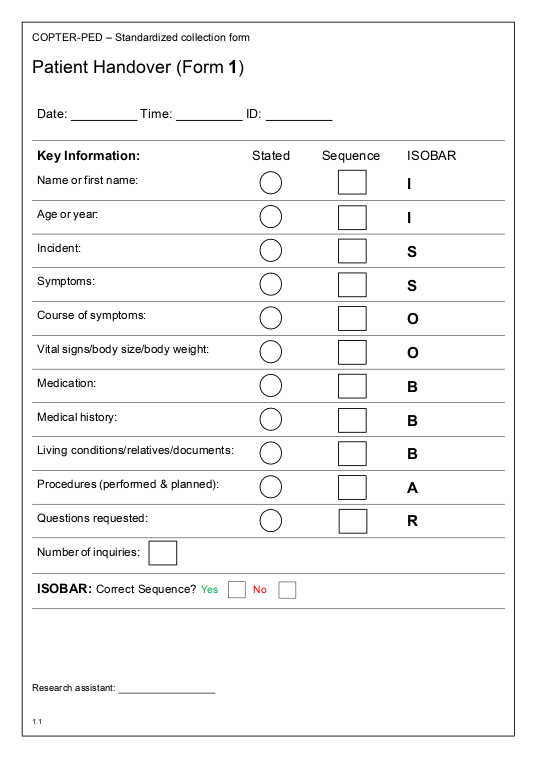


Supplementary Figure S1: Standardized data collection form (1 of 2) for data collection during patient handover. The research assistants noted down the key information transmitted during the handover and its sequence. The number of inquiries during or after the handover was also documented.

## Standardized collection form – Patient Handover (Form 2):


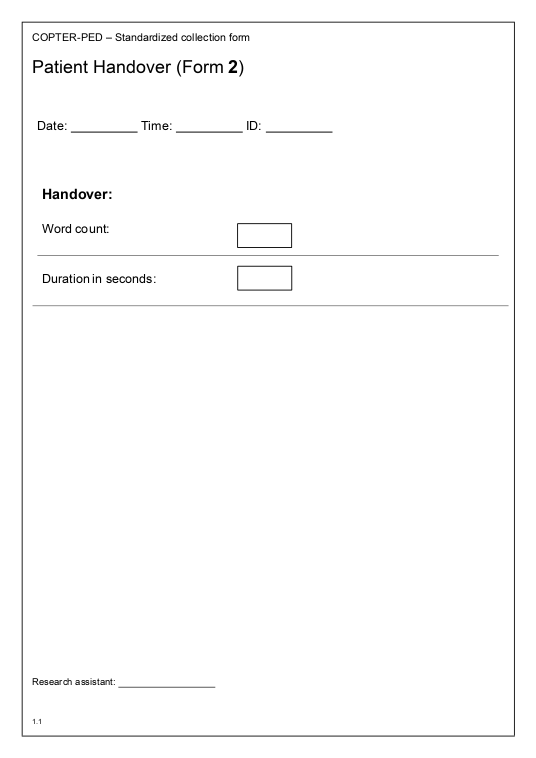


Supplementary Figure S2: Standardized data collection form (2 of 2) for data collection during patient handover. The research assistants counted the words with a hand counter and measured the time of the handover with a stopwatch.

## Standardized collection form – Staff Interview:


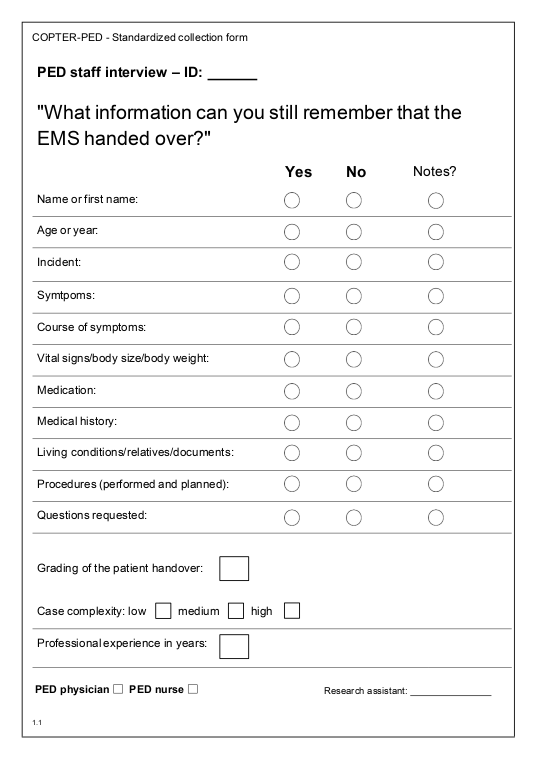


Supplementary Figure S3: Standardized collection form for interviewing ED staff. Fifteen minutes after the handover, the ED staff were asked to repeat the information that they could still remember. They were also asked about their subjective assessment of the handover and the subjective complexity of the case.

# Supplementary Tables

## Primary and Secondary Outcome Measures

|  | | | | | | | | | | **95% CI Mean** | | | |  | | | | | |
| --- | --- | --- | --- | --- | --- | --- | --- | --- | --- | --- | --- | --- | --- | --- | --- | --- | --- | --- | --- |
|  |  | | | **N** | | **Md** | | **M** | | **Upper** | | **Lower** | | **SD** | | **Min** | | **Max** | |
| **KITE** |  | *Baseline* |  | 27 |  | 8.50 |  | 8.43 |  | 8.71 |  | 8.16 |  | 0.14 |  | 6.64 |  | 9.77 |  |
|  |  | *Intervention* |  | 27 |  | 8.81 |  | 8.85 |  | 9.07 |  | 8.62 |  | 0.11 |  | 7.79 |  | 10.00 |  |
|  |  | *Post-Intervention* |  | 26 |  | 8.50 |  | 8.52 |  | 8.81 |  | 8.23 |  | 0.14 |  | 7.22 |  | 9.64 |  |
|  |  | *Final* |  | 27 |  | 8.87 |  | 8.74 |  | 8.97 |  | 8.51 |  | 0.11 |  | 7.64 |  | 9.61 |  |
| **Words** |  | *Baseline* |  | 27 |  | 155 |  | 179.96 |  | 216.48 |  | 143.45 |  | 92.30 |  | 66 |  | 374 |  |
|  |  | *Intervention* |  | 27 |  | 158 |  | 170.70 |  | 206.59 |  | 134.82 |  | 90.71 |  | 51 |  | 386 |  |
|  |  | *Post-Intervention* |  | 26 |  | 182 |  | 206.77 |  | 248.61 |  | 164.93 |  | 103.58 |  | 70 |  | 492 |  |
|  |  | *Final* |  | 27 |  | 132 |  | 166.56 |  | 200.35 |  | 132.76 |  | 85.43 |  | 79 |  | 380 |  |
| **Duration** |  | *Baseline* |  | 27 |  | 78 |  | 84.19 |  | 110.06 |  | 68.31 |  | 40.14 |  | 34 |  | 168 |  |
|  |  | *Intervention* |  | 27 |  | 81 |  | 83.26 |  | 97.47 |  | 69.05 |  | 35.93 |  | 27 |  | 184 |  |
|  |  | *Post-Intervention* |  | 26 |  | 109 |  | 113.54 |  | 135.50 |  | 91.58 |  | 54.38 |  | 37 |  | 245 |  |
|  |  | *Final* |  | 27 |  | 89 |  | 98.37 |  | 115.14 |  | 81.60 |  | 42.40 |  | 29 |  | 203 |  |
| **Key Information** |  | *Baseline* |  | 27 |  | 7 |  | 6.70 |  | 7.49 |  | 5.92 |  | 1.98 |  | 3 |  | 10 |  |
|  |  | *Intervention* |  | 27 |  | 8 |  | 8.19 |  | 8.72 |  | 7.65 |  | 1.36 |  | 5 |  | 10 |  |
|  |  | *Post-Intervention* |  | 26 |  | 9 |  | 8.38 |  | 8.99 |  | 7.78 |  | 1.50 |  | 4 |  | 10 |  |
|  |  | *Final* |  | 27 |  | 8 |  | 8.15 |  | 8.58 |  | 7.71 |  | 1.10 |  | 6 |  | 10 |  |
| **Questions** |  | *Baseline* |  | 27 |  | 2 |  | 1.70 |  | 2.19 |  | 1.22 |  | 1.23 |  | 0 |  | 5 |  |
|  |  | *Intervention* |  | 27 |  | 1 |  | 1.44 |  | 1.86 |  | 1.03 |  | 1.05 |  | 0 |  | 3 |  |
|  |  | *Post-Intervention* |  | 26 |  | 2 |  | 2.85 |  | 3.74 |  | 1.96 |  | 2.20 |  | 0 |  | 9 |  |
|  |  | *Final* |  | 27 |  | 2 |  | 2.07 |  | 3.08 |  | 1.07 |  | 2.54 |  | 0 |  | 13 |  |
| **rKI Nurse** |  | *Baseline* |  | 26 |  | 7.5 |  | 7.31 |  | 7.97 |  | 6.64 |  | 1.64 |  | 3 |  | 10 |  |
|  |  | *Intervention* |  | 26 |  | 9 |  | 8.27 |  | 8.88 |  | 7.66 |  | 1.51 |  | 5 |  | 10 |  |
|  |  | *Post-Intervention* |  | 26 |  | 9 |  | 8.08 |  | 8.74 |  | 7.41 |  | 1.65 |  | 5 |  | 10 |  |
|  |  | *Final* |  | 27 |  | 8 |  | 8.07 |  | 8.50 |  | 7.65 |  | 1.07 |  | 6 |  | 10 |  |
| **rKI Physician** |  | *Baseline*  *Intervention*  *Post-Intervention*  *Final* |  | 20  19  25  27 |  | 7.5  9  8  9 |  | 7.60  8.63  8.44  8.85 |  | 8.37  9.30  8.92  9.27 |  | 6.83  7.97  7.96  8.43 |  | 1.64  1.38  1.16  1.06 |  | 5  5  6  7 |  | 10  10  10  10 |  |
| **Grade Nurse** |  | *Baseline*  *Intervention*  *Post-Intervention*  *Final* |  | 26  26  26  27 |  | 2  2  2  2 |  | 1.98  1.85  1.88  1.87 |  | 2.49  2.12  2.19  2.14 |  | 1.47  1.57  1.58  1.60 |  | 1.27  0.69  0.77  0.67 |  | 1  1  1  1 |  | 6  3  3  3 |  |
| **Grade Physician** |  | *Baseline* |  | 20 |  | 2 |  | 1.80 |  | 2.19 |  | 1.41 |  | 0.83 |  | 1 |  | 4 |  |
|  |  | *Intervention* |  | 19 |  | 1 |  | 1.58 |  | 1.98 |  | 1.18 |  | 0.84 |  | 1 |  | 4 |  |
|  |  | *Post-Intervention* |  | 25 |  | 2 |  | 1.92 |  | 2.33 |  | 1.51 |  | 1.00 |  | 1 |  | 5 |  |
|  |  | *Final* |  | 27 |  | 2 |  | 1.67 |  | 1.94 |  | 1.40 |  | 0.68 |  | 1 |  | 3 |  |

Supplementary Table S1: Primary and secondary outcome measures for all four groups of the trial. Words, Duration (seconds), Key Information, and Questions derive from patient handover conversation.
Abbreviations: Md, median; M, mean; CI, confidence interval; SD, standard deviation; rKI, remembered key information after handover

# References:

1. Nuernberger M, Lang S, Maass T, Lehmann T, Brodoehl S, Lewejohann JC. The Effects of an ISOBAR-Structured Patient Handover Conversation Between Rescue Services and Emergency Department Staff: The COPTER Trial. *JACEP Open*. 2025;6(1):100011. doi:10.1016/j.acepjo.2024.100011
